# Enhanced Electrostatic Safety and Thermal Compatibility of Special Powders Based on Surface Modification

**DOI:** 10.3390/nano14010126

**Published:** 2024-01-04

**Authors:** Xuchao Pan, Libo Zhang, Jialu Guan, Jing Lv, Yifei Xie, Haifeng Yang, Linghua Tan

**Affiliations:** 1 School of Mechanical Engineering, Nanjing University of Science and Technology, Nanjing 210094, China; 2 National Special Superfine Powder Engineering Research Center, Nanjing University of Science and Technology, Nanjing 210094, China; 3 School of Chemistry and Chemical Engineering, Nanjing University of Science and Technology, Nanjing 210094, China; 4 Shanxi Beihua Guanlv Chemical Industry Co., Ltd., Yuncheng 044500, China

**Keywords:** electrostatic accumulation, surface modification strategy, conductive polymer, energetic materials, special powders, thermal stability

## Abstract

Electrostatic accumulation is associated with almost all powder-conveying processes which could bring about electrostatic discharges. In most cases of industrial accidents, electrostatic discharge is proven to be the primary source of ignition and explosion. Herein, a surface modification process of polyaniline (PANI) is proposed to construct highly exothermic special powders, namely, HMX@PANI energetic composites, with low charge accumulation for improving powder electrostatic safety. Pure HMX are encapsulated within the PANI-conductive polymer layer through simple hydrogen bonding. Simulation results demonstrate that the forming process of HMX/aniline structure is a spontaneously thermodynamical process. The resultant inclusion complex exhibits excellent thermal stability, remarkable compatibility and intensive heat release. Importantly, PANI possesses superior electrostatic mobility characteristics because of the π-conjugated ligand, which can significantly reduce the accumulated charges on the surface of energetic powders. Moreover, the modified explosive has a narrower energy gap, which will improve the electron transition by reducing the energy barrier. The electrostatic accumulation test demonstrates that HMX@PANI composites possess a trace electrostatic accumulation of 34 nC/kg, which is two orders of magnitude lower than that of pure HMX (−6600 nC/kg) and might indicate a higher electrostatic safety. In conclusion, this surface modification process shows great promise for potential applications and could be extensively used in the establishment of high electrostatic safety for special powders.

## 1. Introduction

High electrostatic accumulation of powders causes numerous difficulties during the processing, conveying and storage [1,2,3]. Indeed, charged powders could cause damage ranging from losses in quality and productivity to more severe accidents, such as explosions [4]. Especially for special energetic powders with flammable and explosive characteristics, electrostatic discharge can easily lead to accidental explosion accidents, resulting in incalculable losses [5,6,7]. Therefore, there is an urgent need to eliminate the accumulated electrostatic charge of powders to improve product quality and production safety.

Operations like adding conductive materials, humidifying and grounding are often used to suppress the generation of electrostatic charges or accelerate the dissipation of electrostatic charges [8]. Among these solutions, the addition of conductive materials, such as antistatic agents and carbon materials, can inhibit the accumulation of static charges from the perspective of powder material itself and is an effective strategy to improve intrinsic safety [9,10,11]. For instance, carbon materials could be used as conductive additives based on the electron delocalization effect of large π-bonding systems [12]. Li et al. prepared graphene nanosheet–lead styphnate/lead azide composites utilizing graphene nanosheets as conductive fillers, which exhibited satisfying electrostatic spark sensitivities and electrostatic accumulation properties [13]. Lv et al. fabricated the CL-20@GO structure based on the bionic functional strategy, which reduced the electrostatic accumulation of CL-20 [14]. Wang et al. employed graphene (rGO) and carbon nanotubes (CNT) as conductive fillers to minimize the electrostatic accumulation of CL-20-based composites [15]. There is no doubt that substantial results have been attained in previous research on decreasing the electrostatic accumulation of energetic powder by adding conductive carbon additives.

Moreover, conductive polymers, especially for polyaniline (PANI), can also be used as an efficient conductive additive due to its outstanding π-electrons delocalization process and significant electrostatic transport characteristics [16,17]. For this purpose, Lee et al. fabricated carbon nanotube (CNT)/PANI composites by optimizing the PANI content with a simple solution process, which could precisely control the electrical conductivity of the CNT structure [18]. Huang et al. further prepared CNT/PANI film materials with a two-dimensional network structure via the in situ polymerization of PANI on carbon nanotube film [19]. Compared with CNT /PANI powders prepared via a dispersion system, CNT/PANI film possessed excellent electrical conductivity and mechanical properties. Wang et al. employed a straightforward blending technique to construct a skin–core-structured PANI/cellulose network, which exhibited remarkable mechanical strength and electrical conductivity [20]. Additionally, PANI could be deposited onto the surface of various powders to form a stable cladding layer with tight interfacial contact [21]. For example, Yun et al. utilized an interface polymerization method to successfully fabricate the polystyrene (PS)/PANI core–shell microparticles with a narrow particle size distribution [22]. Shao et al. employed a surface chemical grafting approach to in situ polymerize conductive PANI on the surface of fly ashes (FAs) to create the core–shell FAs/PANI microspheres with excellent thermal stability [23]. For energetic materials, Zhang et al. employed the in situ polymerization of polyaniline to effectively reduce the thermal stability and safety of HMX energetic material [24]. Goetz et al. employed an in situ polymerization method to synthesize conductive PANI on the surface of SnO_2_ and form a SnO_2_-PANI/Al nanothermite. The modified structure exhibited a lower spark sensitivity of 47 mJ [25]. In this context, PANI possesses high electrical conductivity and super binding characteristics, which might contribute to reducing electrostatic accumulation toward a high electrostatic safety powder.

Herein, a surface modification process by polymers is proposed to achieve a significant reduction in the electrostatic accumulation of energetic powder. Therein, 1,3,5,7-tetranitro-1,3,5,7-tetraazacyclooctane (HMX), as the most common high-energetic material, is intentionally selected as the embedding explosive [26,27]. PANI-conductive layers are fabricated on the surface of HMX via an in situ polymerization process. The rationality of binding the HMX/Aniline structure is checked out by investigating the electronic energy values (thermal enthalpy, free energy and binding energy) based on the density functional theory. Furthermore, the modified composites exhibit remarkable compatibility and outstanding thermal stability. The simulation results further show that the HMX/aniline structure possesses a higher electron transition capacity due to its narrower band gap. More importantly, the self-polymerized aniline layer ensures that the resultant inclusion complex possesses a lower electrostatic accumulation value of only 34 nC/kg, which might mean a high electrostatic safety of the target product. To sum up, this research might provide a universal strategy for enhancing the electrostatic safety and thermal stability of powders using conductive polymer modification approaches.

## 2. Materials and Methods

### 2.1. Materials

HMX was provided by Baiyin Chemical Industry Co., Ltd., Baiyin, GanSu, China. Aniline (ANI, analytical reagent, 99%) and hydrochloric acid (analytical reagent, 37 wt%) were supplied by Lingfeng Chemical Industry Co., Ltd., Shanghai, China. Ammonium persulfate (APS, analytical reagent, 98%) was purchased from Sinopharm Group Chemical Reagent Co., Ltd., Shanghai, China. Polyvinylpyrrolidone (PVP, analytical reagent, 30%) with a molecular weight of 130,000 was provided by Aladdin Reagents Co., Ltd., Shanghai, China. All reagents were used without any purification.

### 2.2. Preparation of Polymer Modified HMX Composites

In this work, ANI was used as a monomer to synthesize PANI. HMX (7.00 g) and PVP (0.14 g) were dispersed in deionized water (420.0 mL) under stirring for 30 min at room temperature. Subsequently, ANI (0.70 g) and hydrochloric acid (34.9 mL) were added to the above solution via strong mechanical stirring. A deionized water solution containing APS (420.0 mL, 4 mg/mL) was dropwise added into the as-prepared aqueous solution in an ice bath. Meanwhile, the suspension was stirred at 400 rpm for 6 h under 0–5 °C. Finally, the dark green precipitate was collected via centrifugation and washed with DI water and ethanol, subsequently dehydrated at 60 °C for 12 h to obtain the target product in in safe oven, namely, HMX@PANI composites.

### 2.3. Assembly of Home-Made Electrostatic Accumulation Tester

**Scheme 1:**
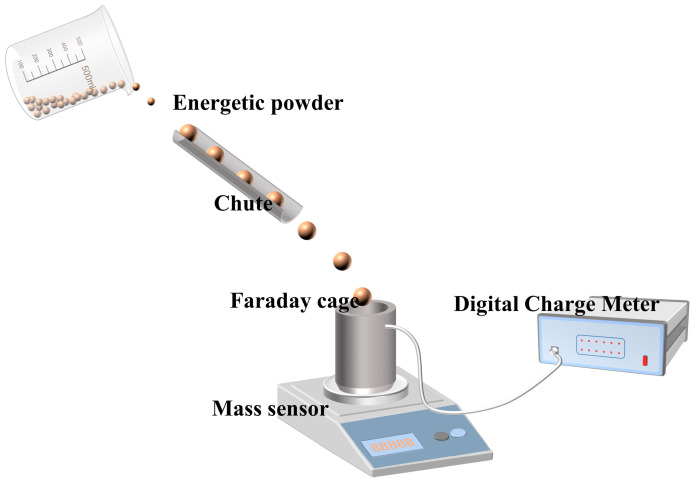
The representation of the electrostatic accumulation tester.

The electrostatic accumulation characteristics of these powders are carried out using an electrostatic accumulation tester by reference to GJB5891.8-2006 method part VIII electrostatic accumulation test. As shown in Scheme 1, the electrostatic accumulation test device mainly includes an automatic feeding system, a chute system and a real-time monitoring system for static electricity and quality. The major component of the chute system is a stainless steel of 100 cm, which is tested with an angle of 45°. The digital charge gauge (ES EST111, Beijing Huajinghui Technology Co., Ltd., Beijing, China) has four test ranges ranging from 10 pC to 20 C and an accuracy of 0.001 nC.

For each test, 5.0 g of sample was poured into the chute and the friction-charged sample was then dropped into a faraday cage. It was noted that the energetic powder needed to be cautiously added to control in an elegant way. The mass-sensor and the digital-charge-meter-recorded mass and electrostatic charge of the specimen fell into the Faraday cage, respectively. Finally, the electrostatic accumulation results were obtained via the charge-to-mass ratio [11].

### 2.4. Characterization Methods

The morphology of pure HMX particles and HMX@PANI composites was observed via a scanning electron microscope (SEM, Phenom G2 Pro, FEI Ltd., Eindhoven, The Netherlands) under an acceleration voltage of 5 kV. The surface chemical composition of samples was studied via X-ray photoelectron spectra (XPS, ESCALAB250Xi, Thermo Fisher Scientific, Waltham, MA, USA). Moreover, high-resolution spectra of XPS were accompanied with a fitting process for photoelectron bands via symmetric Gaussian–Lorentzian and asymmetric Gaussian–Lorentzian line shape, respectively. Before sample analysis, the binding energy scale of XPS equipment was calibrated by C1s line at 284.8 eV. Fourier-transform infrared (FT-IR, NICOLET IS50, Thermo Fisher Scientific, Waltham, MA, USA) spectra were accomplished on a Nicolet Fourier Spectrophotometer-360 using KBr pellets in the range of 400–4000 cm^−1^. The structure characterization was conducted by X-ray diffraction (XRD, D8 Advance, Bruker AXS GmbH, Karlsruhe, Germany) in the 2θ range of 10–50°.

Thermal properties were performed on a differential scanning calorimeter (DSC, SDT-Q600, TA Instruments, New Castle, DE, USA). A roughly 1.0 mg sample was placed in a 40 μL hermetically sealed aluminum pan, and the temperature range was set from 50 to 350 °C with the heating rate of 10 °C/min in argon.

### 2.5. Computational Methods

In this work, ten different and possible HMX/ANI complexes were constructed because of the spatial structure and relative positions of ANI and HMX. The optimized Cartesian coordinates in angstroms and a table of total energies of 10 optimized HMX/ANI complex have been provided in Table S1 and Txt S1 in Supplementary Materials

Meanwhile, the geometric and electronic simulation results of HMX, ANI and all of ten designed HMX/ANI composites were calculated via the M062X/6-31G(d) method [28,29] utilizing the Gaussian 09 software [30]. The energy gap (GAP) of HMX, ANI and all of ten designed HMX/ANI composites was also calculated. The ESP-mapped molecular vdW surface of pure HMX and HMX/ANI complex molecules were calculated with Multiwfn software (version 3.8) [31]. The GaussView 6 software was used to calculate the hydrogen bondings effect in the HMX and ANI systems. Herein, hydrogen bons can be analyzed into strong hydrogen bonds (1.8–2.4 Å), relatively strong hydrogen bonds (2.4–2.8 Å) and weak hydrogen bonds (2.8–3.2 Å) according to their band length.

## 3. Results and Discussion

### 3.1. Morphology and Crystal Structure Characterization

**Figure 1:**
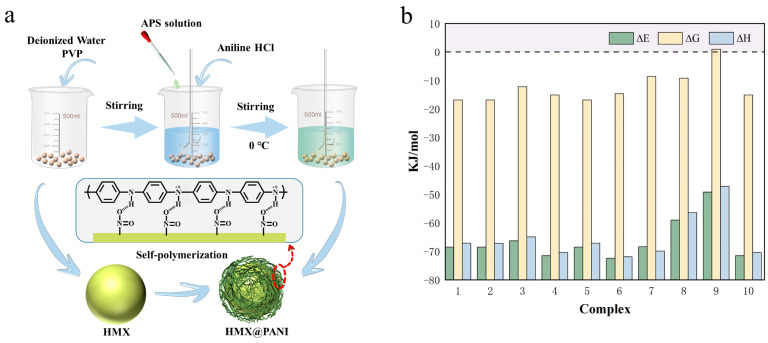
(**a**) Schematic illustration for the encapsulation process of HMX@PANI composites. (**b**) A comparison of the ∆*E*, ∆*G* and ∆*H* values of the 10 designed HMX/ANI complexes.

The encapsulation process of PANI-conductive polymer on the surface of HMX particles is illustrated in Figure 1. First, HMX particles are uniformly dispersed in deionized water with PVP as a stabilizer. When the oxidant APS is added dropwise, the ANI monomer and APS undergo a redox reaction to generate aniline free radicals. In the strong acidic environment, the para-coupled structure is formed and gradually polymerizes into polyaniline to form HMX@PANI structure with a spontaneous encapsulation process on the surface of HMX particles by hydrogen bonding [32].

To predict the possibility of binding ANI with HMX for HMX@PANI composites, the electronic energy values of these optimized structures, including ∆*E*, ∆*G* and ∆*H*, are displayed in Table 1 and Figure 1b. Compared with pure HMX structure, all 10 complexes obviously have negative values of ∆*E*, indicating that the total energy is reduced after the forming of HMX/ANI complexes from independent HMX and ANI molecules. In other words, HMX and ANI can bind together tightly. ∆*H* of all 10 HMX/ANI complexes are also obviously negative, showing that the binding process of HMX and ANI is exothermic, which will also be helpful for forming HMX/ANI complexes. In addition, a comparison for ∆*G* values further shows that most models display negative values and indicates a spontaneously thermodynamical process for the binding of ANI with HMX. Among them, complexes 4, 6 and 10 show the lowest ∆*E*, ∆*H* and ∆*G* values among all complexes, showing that they are the most stable complexes.

**Figure 2:**
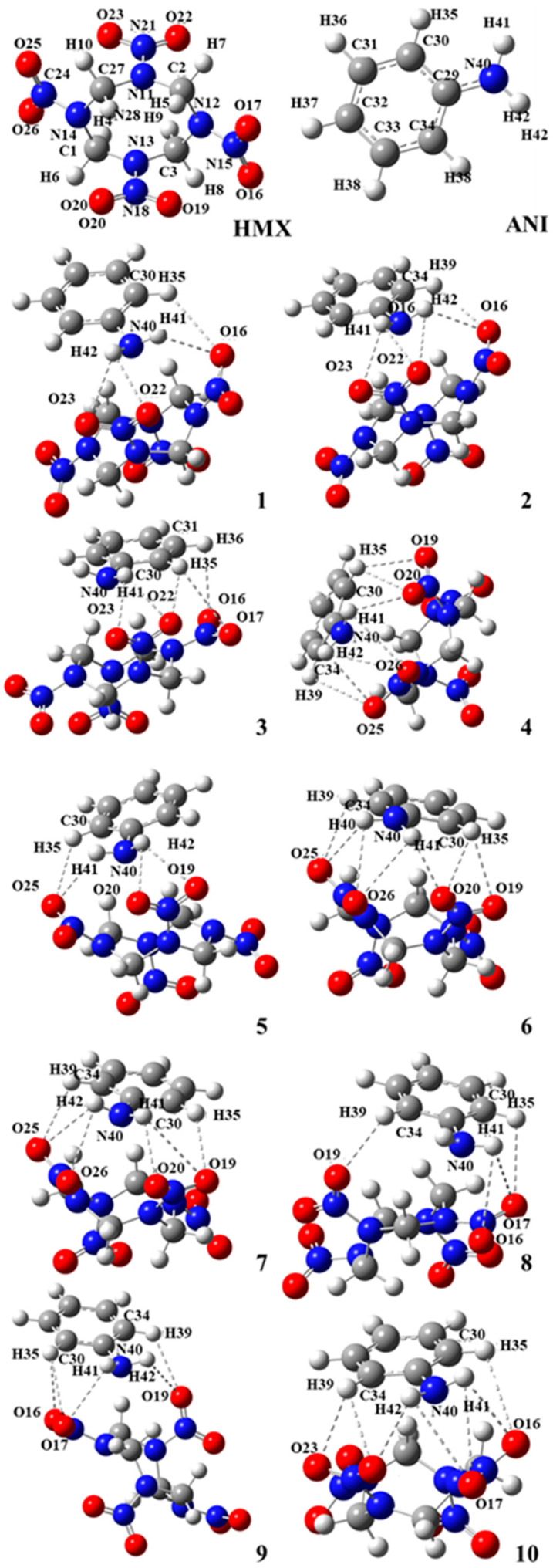
The models of pure HMX, ANI and 10 optimized HMX/ANI complexes with different hydrogen bonding effects. The gray, white, blue and red balls are C, H, N and O atoms, respectively.

The optimized structures of HMX, ANI and ten designed HMX/ANI composites with a hydrogen bonding effect are displayed in Figure 2. Simulated results show that ANI and HMX can be combined through various types of hydrogen bonding to form a stable composite material. Compared with other models, complexes 4, 6 and 10 possess the stronger binding stability due to the strength and number of the hydrogen bondings (Table 2).

**Figure 3:**
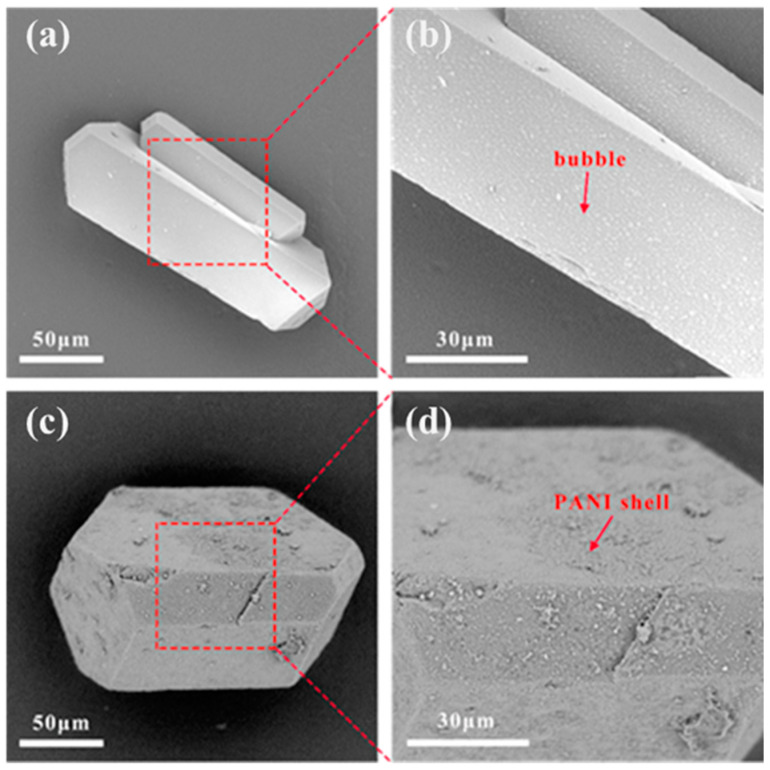
The top-view and magnified view of SEM images for (**a**,**b**) raw HMX particles and (**c**,**d**) HMX@PANI composites.

SEM images of HMX particles exhibit a regular polyhedron morphology with smooth surfaces and sharp edge angles (Figure 3a). Moreover, Figure 3b shows that many white bubbles can be seen in the local magnification of the HMX surface, which is probably caused by the radiation in the electron beam [33,34].

After in situ polymerization, the rougher surface of the HMX@PANI composites compared to the raw HMX crystals demonstrates a successful surface encapsulation process (Figure 3c). Moreover, the magnified view of the SEM image shows that surface of the HMX@PANI composites are without obvious bubbles, which indicates that the PANI-conductive layer is effective in improving the impedance of HMX to the electron beam [34,35].

**Figure 4:**
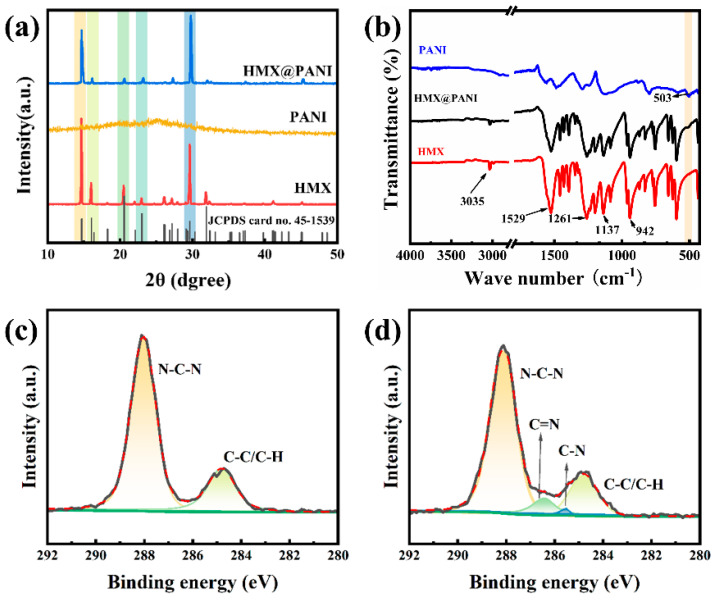
(**a**) XRD patterns, (**b**) FT-IR spectra of raw HMX particles and HMX@PANI composites. High resolution C 1s spectra of (**c**) HMX and (**d**) HMX@PANI composites.

As shown in Figure 4a, the XRD pattern exhibits that the characteristic peaks at 14.6°, 15.9°, 20.4°, 23.0° and 29.5° of the pristine sample correspond to β-form crystal HMX (JCPDS Card No. 45-1539) [26,36]. Moreover, XRD results show that polyaniline is an amorphous polymer. Thus, the strong peak pattern of the HMX@PANI composites in XRD is attributed to the standard cards (PDF# 451539) of β-form crystal HMX. Meanwhile, the intensity of characteristic peaks is slightly weakened compared to raw HMX, which might be due to the covering of PANI layer [37].

As shown in Figure 4b, the FTIR spectra shows that the characteristic peaks at 3035 cm^−1^, 1529 cm^−1^, 1137 cm^−1^, 1261 cm^−1^ and 942 cm^−1^ in the spectra correspond to C-H stretching, -NO_2_ asymmetric stretching, N-N stretching, -NO_2_ stretching symmetric and the ring stretching of HMX, respectively [36]. Remarkably, HMX@PANI composites show a new peak at 503 cm^−1^, corresponding to the aromatic ring bending vibration peak of PANI [38,39]. To sum up, the FTIR results may indicate the successful formation of HMX@PANI composites via the self-polymerization process.

The high-resolution spectra of the C 1s region obtained from HMX crystals and HMX@PANI composites are shown in Figure 4c,d. For raw HMX, the C 1s region is fitted to two peaks at 284.8 eV and 288.0 eV, which are attributed to C-H and N-C-N species [40]. After the encapsulation process of HMX with PANI, XPS spectra shows two new peaks at 285.4 eV and 286.2 eV, corresponding to C-N and C=N, respectively [41]. This result also demonstrates the successful encapsulation of PANI on the HMX surface.

### 3.2. Thermal Behavior

**Figure 5:**
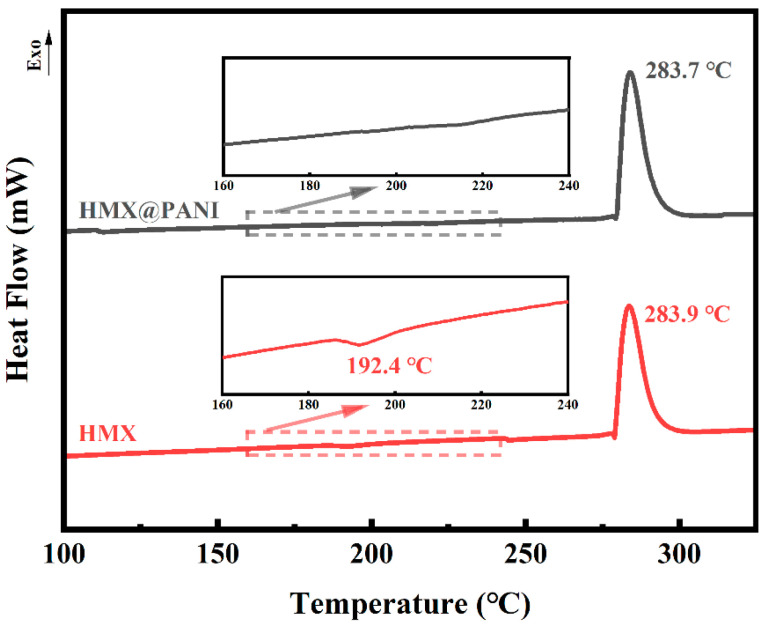
DSC curves of raw HMX particles and HMX@PANI composites.

Figure 5 displays the DSC curves of raw HMX and modified samples. For raw HMX particles, the magnified DSC curve shows a weak endothermic peak at 192.4 °C, corresponding to the phase transition of HMX. According to related literature reports, the thermal decomposition of polyaniline occurs at 250.1 °C, which is lower than the thermal decomposition peak of pure HMX [24,42,43]. However, no obvious thermal decomposition peak of polyaniline is observed in Figure 5, which may be due to the trace addition of polyaniline. It is also consistent with the XRD results. In addition, HMX@PANI composites show no endothermic peak in the inset DSC curves, which are probably attributed to the restriction effect of the PANI covering formed via the in situ polymerization process on the surface of HMX [26,44,45].

It is worth noticing that raw HMX exhibits an intensive heat release process at 275–295 °C, and the exothermic peak temperature is 283.9 °C. By contrast, HMX@PANI composites possess similar thermal decomposition behavior versus raw HMX; meanwhile, the exothermic peak temperature only moves forward by 0.2 °C. In this context, it demonstrates that HMX and the PANI-conductive polymer have remarkable compatibility [46,47].

### 3.3. Electrostatic Accumulation Performance

**Figure 6:**
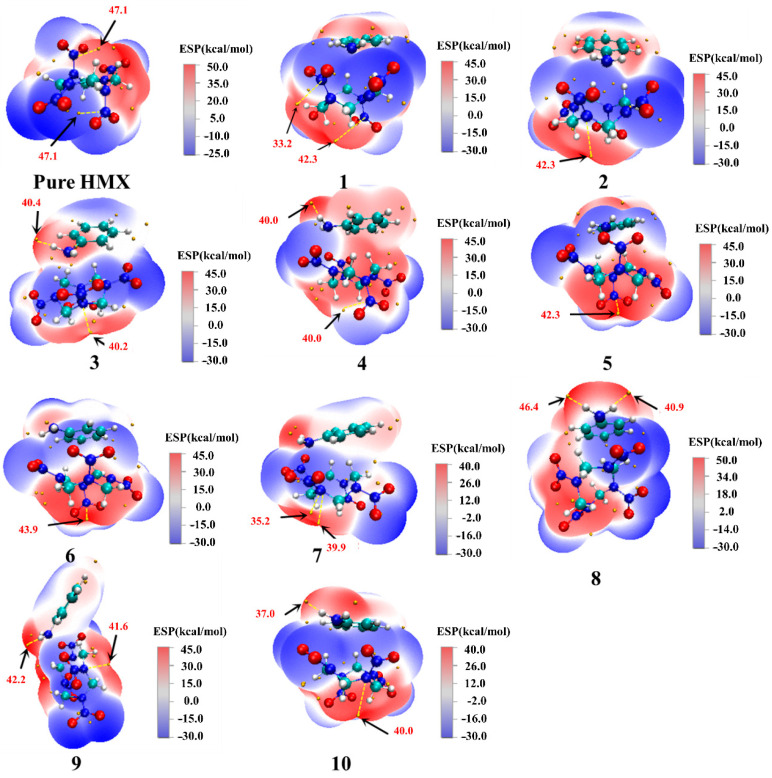
The ESP-mapped molecular v*dW* surface of pure HMX and the 10 designed HMX/ANI composites. The green, white, blue and red balls are C, H, N and O atoms, respectively.

Figure 6 displays a comparison of the area percent in each electrostatic potential (ESP)-mapped molecular v*dW* surface for HMX and the 10 designed HMX/ANI composites. In general, there will be a large value on the negative region and a low value on the positive region for insensitive energetic compounds. During calculation, the value and area of positive ESP of the 10 designed HMX/ANI composites is obviously less than HMX. As a result, these HMX/ANI composites may be more insensitive than pure HMX. Among them, complexes 1, 4 and 7 are the most insensitively structured of the optimized HMX/ANI complexes.

Most energetic compounds are insulators due to their energy gap, making electron transition difficult. More and more electrons are adsorbed and accumulated in the energetic compounds, leading to high electrostatic sensitivity. Thus, if the energy gap could be reduced, the electron transition and electric conductivity of HMX would be promoted, leading to lower electrostatic sensitivity.

**Figure 7:**
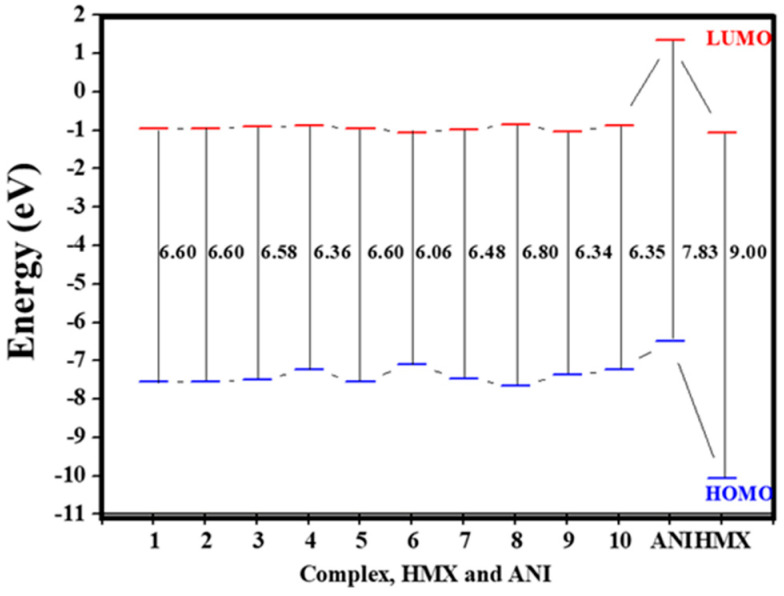
*E*_HOMO_, *E*_LUMO_ and GAP values of HMX, ANI and HMX/ANI composites.

As shown in Figure 7 and Table 3, the E_HOMO_ values of all 10 HMX/ANI complexes are obviously higher than those of HMX, showing that electron donating is much easier. Moreover, the energy gap between the HOMO and LUMO of HMX/ANI complexes is significantly smaller than that of pure HMX. This means that the electron transition and electric conduction of HMX/ANI complexes will be easier than HMX. In other words, less elections will accumulate in the energetic system, leading to lower electrostatic sensitivity. Among them, complex 6 shows a lower energy gap of only 6.06 eV. In this context, complex 6 might form more energy levels to reduce the energy barrier of electron transition, accelerate electron conduction, lower election accumulation and, ultimately, increase the electrostatic safety in the HMX/ANI energetic system. Since the interaction between polymers and energetic compounds is stronger than that between the monomer of polymers and energetic compounds, the energy gap between HMX and PANI may be yet shorter, and the decrease in the sensitivity of HMX by PANI will be more obvious.

To evaluate the electrostatic sensitivity of the above two composites, the electrostatic accumulation data of pure HMX particles and HMX@PANI composites are pointwise collected via an electrostatic accumulation tester. To achieve more accurate experimental data, we repeated the test process for these samples seven times, and electrostatic accumulation values are collected in Table 4.

**Figure 8:**
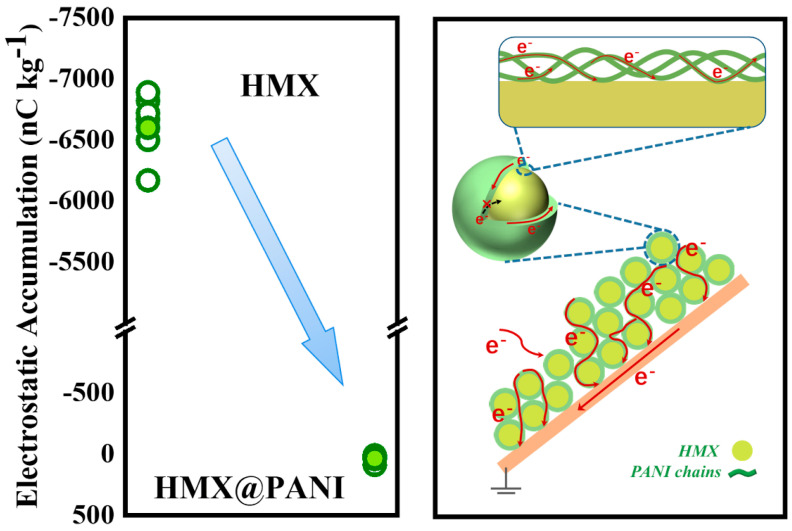
Electrostatic accumulation characteristics of HMX particles and HMX@PANI composites and its schematic of electrostatic accumulation reduction in special powder.

As shown in Figure 8, the average values of electrostatic accumulation for raw HMX and HMX@PANI composites are calculated to be approximately −6600 nC/kg and 34 nC/kg, respectively. The electrostatic accumulation values are decreased by two orders of magnitude, which implies that the presence of the PANI-conductive covering layer significantly reduces the electrostatic accumulation of HMX. Moreover, the HMX@PANI composite is slightly positively charged through friction with the stainless steel chute, which could be attributed to the presence of PANI with a strong electron donor [32,48]. In this context, HMX@PANI composites possess a lower electrostatic accumulation capacity, which could be attributed to two factors: on the one hand, the overlapping orbitals along the polymer backbone contribute to the delocalization of electrons that present twisted polymer chains around the injected charge carriers, leading to the formation of polaritons and their directional movement and resulting in the excellent electrical conductivity of product [21,49]; on the other hand, a continuous conductive matrix might be formed by PANI chains into these powders and enhance the electrostatic transfer process further [50].

## 4. Conclusions

In summary, we present a surface modification process to develop high-safety special powder by encapsulating a labile explosive within conductive polymer. Polyaniline was successfully self-polymerized on the surface of HMX via hydrogen bonding at room temperature. The DFT calculations not only reveal a spontaneous process for binding ANI with HMX; the modified structure can also promote electrons transport with the reducing of the energy gap. The experimental results demonstrate that the polymer framework exhibited outstanding compatibility with HMX, accompanied by a slight temperature rise (only 0.2 °C) for the exothermic decomposition peak. The prepared HMX@PANI composites show higher thermal stability and an intensive heat release process. Furthermore, the PANI shell could evacuate the electrostatic charges on the surface of HMX; thereby, the resultant complex possesses outstanding electrostatic conduction properties. The electrostatic accumulation value of the encapsulated explosive had significantly reduced from −6600 nC/kg to 34 nC/kg versus pure HMX particles. To sum up, the present study not only provides a potential high-safety HMX@PANI energetic composite; it also presents a new insight into the construction of highly electrostatic safety powder materials.

## Figures and Tables

**Table 1:** The predicted ΔE, ΔG and ΔH of designed PANI/HMX complexes.

	ΔE (kJ/mol)	ΔG (kJ/mol)	ΔH (kJ/mol)
Complex 1	−68.5	−16.9	−67.1
Complex 2	−68.5	−16.9	−67.2
Complex 3	−66.3	−12.2	−64.9
Complex 4	71.5	−15.1	−70.4
Complex 5	−68.5	−16.9	−67.1
Complex 6	−72.4	−14.7	−71.9
Complex 7	−68.4	−8.6	−69.9
Complex 8	−59.0	−9.2	−56.4
Complex 9	−49.2	0.9	−47.2
Complex 10	−71.5	−15.1	−70.4

**Table 2:** The strength and number of hydrogen bondings of designed PANI/HMX complexes.

	Stronger Hydrogen Bonds		Strong Hydrogen Bonds		Weak Hydrogen	
	N-H…O	C-H…O	N-H…O	N-H…O	C-H…O	N-H…O
Complex 1	0	0	3	0	0	1
Complex 2	0	0	3	0	1	1
Complex 3	1	0	1	2	0	1
Complex 4	0	0	2	2	2	1
Complex 5	0	0	3	0	0	1
Complex 6	2	0	1	2	1	1
Complex 7	0	0	2	1	2	1
Complex 8	0	0	0	2	2	0
Complex 9	0	0	2	1	0	2
Complex 10	0	0	2	2	2	1

**Table 3:** A comparison in E_HOMO_, E_LUMO_ and GAP values of pure HMX and the 10 designed HMX/ANI composites.

	E_HOMO_ (eV)	E_LUMO_ (eV)	GAP (eV)
ANI	−6.48	1.34	7.83
HMX	−10.07	−1.06	9.00
Complex 1	−7.55	−0.95	6.60
Complex 2	−7.55	−0.95	6.60
Complex 3	−7.49	−0.91	6.58
Complex 4	−7.24	−0.88	6.36
Complex 5	−7.55	−0.95	6.60
Complex 6	−7.11	−1.05	6.06
Complex 7	−7.46	−0.98	6.48
Complex 8	−7.64	−0.84	6.80
Complex 9	−7.37	−1.03	6.34
Complex 10	−7.24	−0.88	6.35

**Table 4:** Electrostatic accumulation values of HMX and HMX@PANI composites.

	Raw HMX	HMX@PANI Composites
1	−6500	11
2	−6820	24
3	−6170	87
4	−6670	39
5	−6890	8
6	−6720	35
7	−6430	34

## Data Availability

The data presented in this study are available on request from the corresponding author.

## Supplementary Materials

The following supporting information can be downloaded at https://www.mdpi.com/article/10.3390/nano14010126/s1: Table S1: Energy information for every configuration; Txt S1: # The following content contains the Cartesian coordinates of atoms in each configuration.
