# Supplementary material for: Enhanced Electrostatic Safety and Thermal Compatibility of Special Powders Based on Surface Modification
Source: Nanomaterials (Basel). 2024 Jan 4;14(1):126. doi: 10.3390/nano14010126 (PMC10781051; doi:10.3390/nano14010126)
Supplement: Supplementary file 1 [file nanomaterials-14-00126-s001.zip › nanomaterials-2764340-supplementary.pdf]

Enhanced Electrostatic Safety and Thermal Compatibility of Special Powders Based on Surface Modification

Xuchao Pan [1], Libo Zhang [2,3], Jialu Guan [2,3], Jing Lv [2,3], Yifei Xie [4], Haifeng Yang [2,3] and Linghua Tan [2,3,*]

1 School of Mechanical Engineering, Nanjing University of Science and Technology, Nanjing 210094, China

2 National Special Superfine Powder Engineering Research Center, Nanjing University of Science and Technology, Nanjing 210094, China

3 School of Chemistry and Chemical Engineering, Nanjing University of Science and Technology, Nanjing 210094, China

4 Shanxi Beihua Guanlv Chemical Industry Co., Ltd., Yuncheng 044500, China

* Correspondence: tanlh@njust.edu.cn

Table S1. Energy information for every configuration.

	ΔE(eV)	ΔH(eV)	ΔG(eV)
HMXAE1	-1483.244420	-1483.243476	-1483.322573
HMXAE2	-1483.244424	-1483.243480	-1483.322560
HMXAE3	-1483.243579	-1483.242634	-1483.320761
HMXAE4	-1483.245666	-1483.244722	-1483.321881
HMXAE5	-1483.244412	-1483.243468	-1483.322563
HMXAE6	-1483.246239	-1483.245295	-1483.321743
HMXAE7	-1483.245467	-1483.244523	-1483.319395
HMXAE8	-1483.240343	-1483.239399	-1483.319647
HMXAE9	-1483.236816	-1483.235872	-1483.315800
HMXAE10	-1483.245665	-1483.244721	-1483.321872

Txt S1

# The following content contains the Cartesian coordinates of atoms in each configuration.

HMXAE1

0 1

C	0.54403400	-1.21380100	-0.38886400
C	1.43973500	2.07912300	0.16070400
C	-0.11030900	0.95408200	-1.54044100
H	-0.39021300	-0.94305000	0.10508200
H	2.34275400	1.91964900	-0.43247400
H	0.44439600	-2.18291000	-0.87611100
H	1.48466700	3.04711700	0.65657900
H	-0.12023200	1.24659900	-2.59018400
H	-1.10190400	0.62255100	-1.23604000
H	2.59674700	-0.36441800	2.14702500
N	1.39177100	0.99938800	1.13834700
N	0.29436100	2.10598100	-0.71540500
N	0.79194900	-0.14579300	-1.35403700
N	1.59069600	-1.33977600	0.60318600
N	-0.66217700	3.06750300	-0.53352600
O	-1.72690700	2.86582800	-1.10529200
O	-0.38200500	4.04510600	0.12438900
N	1.94543600	-0.17783800	-2.09079900
O	2.19006800	0.79979400	-2.77634200
O	2.63980600	-1.16952300	-1.96370500
N	0.49085800	1.11633000	2.16600000
O	-0.10117300	2.17386200	2.25159200
O	0.34463300	0.14255700	2.88138300
N	2.43916600	-2.42920700	0.48303500
O	3.52818600	-2.30461700	1.01317900
O	2.01760500	-3.41597000	-0.07770100
C	2.26362200	-0.14481400	1.13413100
H	3.13525500	0.10370800	0.51902500
C	-2.80101400	-0.46868000	0.48819600
C	-3.51248200	-0.35364800	-0.71147700
C	-4.09577300	-1.47901500	-1.28651100
C	-3.97749100	-2.72804700	-0.68269900
C	-3.26659300	-2.84188000	0.51050100
C	-2.67911400	-1.72542500	1.09440600
H	-3.61076800	0.62309800	-1.18100300

H	-4.65038000	-1.37382200	-2.21417500
H	-4.43447200	-3.60190000	-1.13437800
H	-3.16573100	-3.80905400	0.99362800
H	-2.11516200	-1.81596000	2.02068700
N	-2.11143000	0.63687700	1.01742700
H	-2.51628300	1.52945900	0.74772100
H	-2.00246300	0.60103400	2.02733300

HMXAE2

0 1

C	0.54286700	-1.21405000	-0.38873200
C	1.44238300	2.07788700	0.16078400
C	-0.10937300	0.95470700	-1.53994100
H	-0.39100700	-0.94244700	0.10544900
H	2.34496300	1.91736400	-0.43276600
H	0.44223000	-2.18298500	-0.87610300
H	1.48861800	3.04578100	0.65671200
H	-0.11900500	1.24741500	-2.58963600
H	-1.10131000	0.62425900	-1.23548900
H	2.59789700	-0.36713100	2.14624600
N	1.39369700	0.99808000	1.13844100
N	0.29663900	2.10595600	-0.71468600
N	0.79167000	-0.14619800	-1.35381500
N	1.58972800	-1.34120400	0.60301100
N	-0.65851000	3.06884000	-0.53294300
O	-1.72355900	2.86850700	-1.10450900
O	-0.37689300	4.04620100	0.12474700
N	1.94481000	-0.17976200	-2.09109300
O	2.19052900	0.79758500	-2.77665900
O	2.63784900	-1.17241400	-1.96435500
N	0.49271600	1.11533500	2.16582400
O	-0.09895400	2.17309100	2.25130600
O	0.34591400	0.14161900	2.88124300
N	2.43685300	-2.43167500	0.48255000
O	3.52632800	-2.30825500	1.01202300
O	2.01377700	-3.41801200	-0.07776300
C	2.26432400	-0.14706000	1.13360300
H	3.13582000	0.10064200	0.51797300
C	-2.80167200	-0.46651700	0.48831100
C	-2.68107900	-1.72366600	1.09388700
C	-3.27068900	-2.83905600	0.51007200
C	-3.98240000	-2.72365200	-0.68248600

C	-4.09932800	-1.47420400	-1.28570300
C	-3.51391500	-0.34991300	-0.71073600
H	-2.11648500	-1.81531100	2.01967700
H	-3.17079100	-3.80655400	0.99274200
H	-4.44107200	-3.59658300	-1.13423900
H	-4.65449500	-1.36791700	-2.21290900
H	-3.61118600	0.62716400	-1.17977600
N	-2.10996500	0.63783600	1.01751500
H	-2.00042300	0.60138000	2.02732100
H	-2.51385100	1.53103800	0.74855200

HMXAE3

0 1

C	-1.20440700	0.92198200	-0.74719000
C	-0.24572700	-1.97099200	0.75126600
C	0.54761600	-0.80879200	-1.38173300
H	-0.30379900	1.28591500	-0.24951700
H	-0.98760000	-2.52316800	0.16557900
H	-1.57353400	1.64965400	-1.46851100
H	0.21770800	-2.62649000	1.48568900
H	0.69251900	-1.48750900	-2.22148300
H	1.22888300	0.04233200	-1.47315400
H	-2.86183800	-0.25911100	1.96656500
N	-0.96262200	-0.90278100	1.41781300
N	0.81834000	-1.48356100	-0.10194000
N	-0.80792400	-0.32682700	-1.39164500
N	-2.24457000	0.74991700	0.25288800
N	2.04276500	-2.09726200	-0.02214900
O	2.77003600	-1.94168700	-0.98746200
O	2.31209600	-2.71635000	0.98848100
N	-1.77368400	-1.08977500	-1.99439600
O	-1.45777600	-2.20896300	-2.35916700
O	-2.87909300	-0.58648800	-2.07858700
N	-0.34421000	-0.29047500	2.47986400
O	0.79080400	-0.65887300	2.72556400
O	-0.96883100	0.56783700	3.06706500
N	-3.50971900	1.20823300	-0.09438600
O	-4.44062200	0.67668700	0.48011900
O	-3.57873500	2.12283600	-0.88582100
C	-2.28790400	-0.47330900	1.06886900
H	-2.77336300	-1.28667600	0.51828400
C	2.02046400	1.87616500	0.50420900

C	2.98256200	0.86737400	0.63839900
C	3.93145300	0.67316300	-0.35946500
C	3.93254200	1.46369800	-1.50797100
C	2.97234000	2.46267400	-1.64465300
C	2.01904400	2.66929700	-0.65093300
H	2.97160600	0.23338200	1.52255100
H	4.66606500	-0.11708800	-0.24310700
H	4.67399000	1.30352400	-2.28302900
H	2.96217300	3.09173100	-2.52984100
H	1.27457400	3.45524900	-0.76031000
N	1.00229700	2.01791000	1.45832400
H	1.24099000	1.65288400	2.37538500
H	0.63598900	2.96016100	1.53795700

HMXAE4

0 1

C	-0.33897800	-1.94065000	1.04522400
C	1.18342100	0.44568500	-0.81278100
C	1.72652000	-0.48621400	1.47013000
H	0.44748100	-2.66567500	0.81403700
H	0.20287400	0.90349300	-0.66742700
H	-1.03919100	-2.35962400	1.76479000
H	1.73900500	0.99449100	-1.57155800
H	2.12349500	-0.09182400	2.40134200
H	2.24622800	-1.41964500	1.22577700
H	-0.43315000	-2.26946300	-2.06440800
N	0.89942300	-0.92734000	-1.22155800
N	1.94056700	0.52337100	0.42296600
N	0.32791800	-0.78373600	1.61705300
N	-1.09268300	-1.62859900	-0.14732400
N	3.27304400	0.90328600	0.29970000
O	4.01463300	0.53118100	1.18861300
O	3.57081700	1.60657700	-0.63994800
N	-0.43525800	0.10601900	2.33844200
O	0.13701500	1.07148000	2.80257100
O	-1.61863700	-0.15653200	2.43969300
N	1.93322500	-1.81272300	-1.35921000
O	3.05361800	-1.34292800	-1.27944900
O	1.65044900	-2.98859800	-1.51338400
N	-2.42664900	-1.92403900	-0.20655500
O	-2.93643500	-1.79733500	-1.30955500
O	-2.98890900	-2.27750000	0.80691600

C	-0.44794500	-1.37505600	-1.44159000
H	-0.99938800	-0.58015600	-1.94432100
C	-2.38249300	1.59334600	-0.32311700
C	-1.57907500	2.37012500	0.52875600
C	-0.62900000	3.24022300	0.00333000
C	-0.45688600	3.36613300	-1.37496200
C	-1.25406400	2.59909900	-2.22443000
C	-2.20015700	1.71558400	-1.71264800
H	-1.71292300	2.29336000	1.60433700
H	-0.01941400	3.82600400	0.68497500
H	0.27999200	4.05182400	-1.77886900
H	-1.14232100	2.68810000	-3.30147400
H	-2.82007500	1.12091700	-2.38046200
N	-3.28744600	0.68221200	0.18932800
H	-3.51542400	0.76912000	1.17004700
H	-4.07064600	0.42952000	-0.39779600

HMXAE5

0 1

C	-1.43943200	2.07873600	0.16170300
C	-0.54402800	-1.21404900	-0.38963900
C	-2.26241300	-0.14544200	1.13475300
H	-2.34300000	1.91932500	-0.43064300
H	0.39049000	-0.94347400	0.10387900
H	-1.48411600	3.04638400	0.65827400
H	-0.44478600	-2.18294600	-0.87736800
H	-2.59481300	-0.36523000	2.14783900
H	-3.13441300	0.10347100	0.52032900
H	0.12005000	1.24700100	-2.59029800
N	-0.79229200	-0.14556900	-1.35435800
N	-1.59009900	-1.34028900	0.60290600
N	-1.38991500	0.99830300	1.13850300
N	-0.29518300	2.10660900	-0.71587500
N	-2.43884500	-2.42952400	0.48275800
O	-3.52747500	-2.30498300	1.01370300
O	-2.01779300	-3.41607100	-0.07870400
N	-0.48985200	1.11578500	2.16679800
O	-0.34377000	0.14214700	2.88240600
O	0.10180400	2.17348600	2.25261900
N	-1.94634700	-0.17703300	-2.09025100
O	-2.64083300	-1.16861900	-1.96312500
O	-2.19126200	0.80101000	-2.77511900

N	0.66167700	3.06793000	-0.53382000
O	1.72620800	2.86632300	-1.10579400
O	0.38173300	4.04541400	0.12441300
C	0.10976700	0.95449800	-1.54055300
H	1.10130600	0.62299400	-1.23591600
C	2.80116600	-0.46881100	0.48778300
C	3.51240100	-0.35365700	-0.71198900
C	4.09527600	-1.47908200	-1.28734800
C	3.97680600	-2.72817300	-0.68369300
C	3.26614800	-2.84206200	0.50963300
C	2.67903300	-1.72555500	1.09384300
H	3.61084300	0.62314800	-1.18132500
H	4.64971700	-1.37392900	-2.21511200
H	4.43349000	-3.60204300	-1.13564200
H	3.16517100	-3.80929500	0.99260400
H	2.11518200	-1.81610400	2.02017200
N	2.11184000	0.63692600	1.01739000
H	2.51668800	1.52935600	0.74728900
H	2.00404800	0.60131600	2.02742600

HMXAE6

0 1

C	-0.19803600	-1.92790100	1.07638300
C	1.20002300	0.50098300	-0.81596500
C	1.78672100	-0.35871300	1.48391700
H	0.62419900	-2.61393300	0.85397000
H	0.19655800	0.90835900	-0.67208400
H	-0.87506500	-2.37262500	1.80285000
H	1.72128200	1.07069900	-1.58372600
H	2.15251000	0.07793600	2.40947300
H	2.35718200	-1.26914500	1.26790400
H	-0.28401800	-2.29903000	-2.04158300
N	0.98659200	-0.89033900	-1.20984900
N	1.95613900	0.63445800	0.41378900
N	0.40387400	-0.72626000	1.62558900
N	-0.96456500	-1.68091000	-0.12556800
N	3.27289700	1.06649000	0.28082000
O	4.02660200	0.74455900	1.17885900
O	3.54234500	1.75869200	-0.67460000
N	-0.41725600	0.14622800	2.30354100
O	0.09280700	1.15123500	2.74912000
O	-1.58818900	-0.17840700	2.38890100

N	2.05939600	-1.73256900	-1.32426700
O	3.15721300	-1.21330200	-1.24797300
O	1.82677500	-2.92223600	-1.45619500
N	-2.29534100	-1.96953900	-0.15901600
O	-2.83113300	-1.83962300	-1.25266300
O	-2.84746800	-2.31212700	0.86584000
C	-0.33648200	-1.40129300	-1.42586500
H	-0.92774700	-0.63327200	-1.92666300
C	-2.58234200	1.43417700	-0.30153200
C	-1.77446900	2.27849500	0.48404500
C	-0.85199100	3.13138700	-0.11130700
C	-0.69802300	3.16791600	-1.49812900
C	-1.47895200	2.31792600	-2.28030700
C	-2.40187800	1.45247100	-1.69739500
H	-1.90243400	2.28050400	1.56293800
H	-0.24717700	3.77404700	0.52198100
H	0.01640100	3.84307000	-1.95697400
H	-1.37736700	2.32947200	-3.36215400
H	-3.02129100	0.80945900	-2.31854100
N	-3.57721100	0.67418300	0.27831500
H	-3.44756300	0.42507700	1.25016800
H	-3.97617600	-0.06582900	-0.28443900

HMXAE7

0 1

C	-0.62130900	-2.14835100	0.61834100
C	1.25780300	0.43868100	-0.78486200
C	1.46716500	-0.89934400	1.35105000
H	0.15465000	-2.86047100	0.32802100
H	0.38782500	1.08632100	-0.68584000
H	-1.35498200	-2.63333700	1.25850300
H	1.99980000	0.91123400	-1.42711600
H	1.81621800	-0.71306000	2.36291400
H	1.92478600	-1.82465300	0.98059500
H	-0.69559600	-1.60418900	-2.59482100
N	0.76528500	-0.80328600	-1.36003800
N	1.87715000	0.24120000	0.52072900
N	0.03306100	-1.06498300	1.34373500
N	-1.30616800	-1.73003900	-0.58123700
N	3.24809600	0.46644100	0.56650900
O	3.85949300	-0.12849700	1.43387500
O	3.70857200	1.26961700	-0.21577300

N	-0.65530900	-0.49216000	2.40131600
O	-0.07307900	0.38310800	3.01273700
O	-1.77899800	-0.89472600	2.61547000
N	1.67266000	-1.78562700	-1.65414500
O	2.84222000	-1.51371500	-1.44928200
O	1.22947700	-2.84861100	-2.05132000
N	-2.66443500	-1.56382500	-0.55383000
O	-3.14489800	-0.99816700	-1.52665000
O	-3.28357200	-2.01131600	0.38607400
C	-0.61817400	-1.02348900	-1.67595400
H	-1.08443400	-0.04762700	-1.81307400
C	-1.79903600	2.01626600	0.03757700
C	-0.81671600	2.65094600	0.81190800
C	0.07404800	3.54089100	0.22077900
C	0.00810200	3.81859100	-1.14404300
C	-0.98017400	3.20429500	-1.91122600
C	-1.88060200	2.31462600	-1.33180200
H	-0.73964600	2.42174700	1.87124800
H	0.83455200	4.01327300	0.83525200
H	0.70944400	4.50975800	-1.59862700
H	-1.05757100	3.42016800	-2.97286100
H	-2.65045300	1.83534500	-1.93343500
N	-2.60461300	1.01969500	0.58889300
H	-2.74516300	1.07741300	1.59140000
H	-3.47988500	0.85596200	0.10266500

HMXAE8

0 1

C	-2.16840700	0.13245700	-1.21497400
C	0.58623000	0.80422800	0.52350400
C	0.27203700	-0.10012300	-1.83001000
H	-1.88224200	1.13202800	-1.55165900
H	0.71695100	-0.23648400	0.83303300
H	-2.94201900	-0.27357400	-1.86689700
H	1.34236100	1.42409400	0.99594800
H	1.01339500	-0.89437100	-1.93469200
H	0.08080200	0.35837500	-2.80099000
H	-2.38609700	0.68892200	2.11355000
N	-0.73275800	1.21472000	0.96422700
N	0.74537300	0.94406500	-0.91486200
N	-0.94050100	-0.65444600	-1.27180100
N	-2.72832400	0.21847100	0.11631300

N	1.80894300	1.67680600	-1.39627500
O	1.95670300	1.66605300	-2.60433300
O	2.50108500	2.27731200	-0.59986000
N	-1.05579000	-2.01579800	-1.11262800
O	-0.09682500	-2.71172800	-1.37999900
O	-2.12994100	-2.40126500	-0.68933300
N	-1.01228300	2.56612500	0.92714000
O	-0.10206900	3.29164300	0.57841000
O	-2.13694100	2.90065100	1.24101000
N	-3.85924900	-0.55028800	0.35979600
O	-4.03458500	-0.88197300	1.51583800
O	-4.59181600	-0.77919500	-0.57727600
C	-1.81558200	0.32087300	1.26473100
H	-1.38036700	-0.65435200	1.50979600
C	3.22622000	-0.98509800	0.06361400
C	3.58978900	-0.15010600	1.12780000
C	3.18283000	-0.45251500	2.42340600
C	2.41494000	-1.58586600	2.68446100
C	2.07538700	-2.43220500	1.62867300
C	2.47835700	-2.14051200	0.32927800
H	4.16896500	0.74755800	0.92886900
H	3.47115600	0.20713800	3.23653100
H	2.10157700	-1.81672500	3.69705600
H	1.49252900	-3.33013200	1.81286400
H	2.19897600	-2.79844000	-0.48875700
N	3.50605200	-0.62734500	-1.26107800
H	4.21403100	0.09172000	-1.36305000
H	3.69404300	-1.41070600	-1.87599000

HMXAE9

0 1

C	-2.01317400	-0.74148300	-1.32190500
C	-0.42612100	1.21895000	0.91248800
C	0.44209900	-0.21556300	-0.96764100
H	-1.83858400	0.13478800	-1.95098800
H	-0.25650200	0.34028100	1.54008200
H	-2.33603400	-1.58203300	-1.93527000
H	-0.06772300	2.11168800	1.41825800
H	1.32765400	-0.73707200	-0.59771000
H	0.51807900	-0.07997700	-2.04631700
H	-3.73761300	0.65833600	1.24146800
N	-1.85332900	1.31564100	0.67267100

N	0.29859500	1.10757700	-0.33830700
N	-0.72978500	-1.00054100	-0.67598300
N	-3.08340100	-0.45907700	-0.38585300
N	1.41033400	1.90773000	-0.50624000
O	2.10458300	1.63295200	-1.46617700
O	1.59779900	2.81810000	0.27628800
N	-0.66313200	-2.00033100	0.25709400
O	0.35939400	-2.09667500	0.91368500
O	-1.65843900	-2.69266100	0.36778900
N	-2.31077300	2.48787700	0.10042800
O	-1.48342600	3.36155600	-0.05981000
O	-3.48946000	2.52878200	-0.18894600
N	-4.03840600	-1.46103000	-0.23874500
O	-4.64080200	-1.46522800	0.81647300
O	-4.22384400	-2.20234000	-1.17841700
C	-2.79827200	0.25616800	0.87157500
H	-2.38232500	-0.42947400	1.61821900
C	3.44749500	-0.32599000	0.87588500
C	4.32089100	0.57094900	0.25293100
C	5.32038100	0.09810300	-0.58793800
C	5.46466900	-1.26776300	-0.82338700
C	4.59110500	-2.15894500	-0.20746500
C	3.58643100	-1.69727400	0.63813900
H	4.19154200	1.63952700	0.41011900
H	5.98963400	0.80695200	-1.06579900
H	6.24720200	-1.63157400	-1.48080100
H	4.69087900	-3.22660900	-0.37920700
H	2.90586200	-2.39637400	1.11740000
N	2.38567200	0.15818800	1.67375600
H	2.62149300	1.02882900	2.14093200
H	2.07656200	-0.52834400	2.35471100

HMXAE10

0 1

C	-1.18316500	0.44618200	-0.81257800
C	0.33856800	-1.94096700	1.04485600
C	0.44722600	-1.37535400	-1.44196600
H	-0.20237300	0.90342400	-0.66720300
H	-0.44809000	-2.66579900	0.81369400
H	-1.73846500	0.99547800	-1.57120700
H	1.03881300	-2.36021300	1.76424900
H	0.43174500	-2.26996100	-2.06448500

H	0.99919700	-0.58112200	-1.94521300
H	-2.12352800	-0.09179100	2.40134100
N	-0.32808700	-0.78413600	1.61723200
N	1.09205700	-1.62894500	-0.14776700
N	-0.89989900	-0.92690200	-1.22183700
N	-1.94013000	0.52398000	0.42321300
N	2.42605800	-1.92426600	-0.20718700
O	2.93563500	-1.79770800	-1.31021900
O	2.98847100	-2.27754800	0.80626900
N	-1.93414700	-1.81175800	-1.35953700
O	-1.65201500	-2.98776000	-1.51386300
O	-3.05432400	-1.34141800	-1.27966600
N	0.43525000	0.10514100	2.33907400
O	1.61848400	-0.15786600	2.44052200
O	-0.13674300	1.07071200	2.80335000
N	-3.27274100	0.90408600	0.29982300
O	-4.01450300	0.53180800	1.18846700
O	-3.57016400	1.60771600	-0.63962900
C	-1.72653600	-0.48604300	1.47009200
H	-2.24652200	-1.41921100	1.22537000
C	2.38310500	1.59283600	-0.32280600
C	2.20116300	1.71515400	-1.71240100
C	1.25547200	2.59900400	-2.22434100
C	0.45831700	3.36621000	-1.37499400
C	0.63012200	3.24025500	0.00332400
C	1.57987000	2.36987600	0.52892900
H	2.82087500	1.12006800	-2.38005500
H	1.14393400	2.68803400	-3.30140700
H	-0.27827300	4.05211600	-1.77905700
H	0.02054500	3.82616600	0.68487700
H	1.71353400	2.29303200	1.60453300
N	3.28729700	0.68107600	0.18983500
H	4.07087100	0.42825000	-0.39673400
H	3.51454100	0.76718000	1.17080400
